# Oral bioaccessibility of potentially toxic elements in various urban environmental media

**DOI:** 10.1007/s10653-024-02073-5

**Published:** 2024-06-20

**Authors:** Martin Gaberšek, Mateja Gosar

**Affiliations:** Geological Survey of Slovenia, Dimičeva Ulica 14, 1000 Ljubljana, Slovenia

**Keywords:** Urban geochemistry, UBM, Soil, Attic dust, Street dust, Household dust

## Abstract

An important aspect of geochemical studies is determining health hazard of potentially toxic elements (PTEs). Key information on PTEs behaviour in the human body in case of their ingestion is provided with the use of in vitro bioaccessibility tests. We analysed and compared oral bioaccessibility of a wide range of PTEs (As, Cd, Ce, Cr, Cu, Hg, La, Li, Ni, Pb, Sb, Sn, Zn), including some that are not often studied but might pose a human health hazard, in soil, attic dust, street dust, and household dust, using Unified BARGE Method (UBM). Additionally, feasibility of usage of scanning electron microscope techniques in analyses of solid residuals of UBM phases was tested. Results show that bioaccessible fractions (BAFs) of PTEs vary significantly between individual samples of the same medium, between different media and between the gastric and gastro-intestinal phases. In soil, attic dust and street dust, bioaccessibility of individual PTE is mostly higher in gastric than in gastro-intestinal phase. The opposite is true for PTEs in household dust. In all four media, with the exception of Pb in household dust, among the most bioaccessible PTEs in gastric phase are Cd, Cu, Pb, and Zn. During the transition from the stomach to small intestine, the mean BAFs of most elements in soil, attic dust, and street dust decreases. The most bioaccessible PTEs in gastro-intestinal phase are Cu, Cd, Ni, and As. Micromorphological and chemical characterisation at individual particle level before and after bioaccessibility test contribute significantly to the understanding of oral bioaccessibility.

**Supplementary Information:**

The online version contains supplementary material available at 10.1007/s10653-024-02073-5.

## Introduction

An important aspect of the geochemical studies of urban environment is determining potential health hazard of potentially toxic elements (PTEs) present in various environmental media. Data on total levels of PTEs alone is not enough for reliable risk assessment as it does not tell us anything about chemical/mineral phases of PTEs and how they behave in the contact with human body. Key information on PTEs behaviour in the human body in a case of their ingestion or inhalation can be provided through bioavailability and bioaccessibility studies. These two terms are often not clearly defined in the literature or they are mistakenly used as synonyms. Bioaccessibility is defined as the maximum amount (e.g., in mg/kg) or the proportion (in %) of a given PTE that is available for potential passage from the digestive tract and lungs into the human bloodstream (Hamel et al., 1998; Ruby et al., 1996; Wragg et al., 2011), while bioavailability is the amount or the proportion of a PTE that actually enters the human bloodstream or it crosses cell membranes (Hamel et al., 1998; Heaney, 2001), which may have negative impacts on human health. Determination of the bioavailability of ingested PTEs is based on research on living organisms (in vivo methods), such as juvenile pigs, primates, rabbits, and rodents (Juhasz et al., 2007). These methods are expensive, time-consuming, and often ethically questionable. That’s why in vitro methods for determining bioaccessibility, which mimic the physiological properties of the human organism in the laboratory, are more appropriate and attainable in environmental geochemical research. With these methods, physiological properties and processes like pH values, body temperature, presence of enzymes, peristalsis, and retention time are simulated.

The oral exposure of humans to PTEs is a well-known fact. Due to the different physiological characteristics of the developing body and their specific behaviour (e.g., hand-to-mouth activity), children are particularly vulnerable group (Moya et al., 2004). On average, children can consume between 39 and 271 mg of soil per day (Moya et al., 2004). Average indoor hand-to-mouth behaviour range from 6.7 to 28.0 contacts/hour, with the lowest value corresponding to the 6 to < 11 years olds and the highest value corresponding to the 3 to < 6 month olds (Xue et al., 2007). The oral bioaccessibility of PTEs depends on many factors, both on the physico-chemical properties of the ingested solid particles and on the physico-chemical conditions in the digestive tract. One of the most important factors of bioaccessibility are chemical/mineral phases in which ingested PTEs occur. In soil, these phases depend on pH, redox potential, cation exchange capacity, contents of organic matter, Fe, Mn and Al-oxides and hydroxides, soil texture, bacterial activity, and humidity (Kabata Pendias, 2011; Rieuwerts et al., 1998; Tack, 2010; Walraven et al., 2015). The pH values of digestive fluids are also a crucial factor in bioaccessibility. The size of the ingested particles is also of vital importance, since smaller particles are usually more bioaccessible as larger particles, due to their larger specific surface area (Ruby et al., 1999).

The presented complexity of oral bioaccessibility led to development of many different methods for its determination in the last 30 years (e.g. PBET method (Ruby et al., 1996), IVG (Rodriguez et al., 1999), SBRC (Juhasz et al., 2009), etc.). These methods differ from each other in the number of steps (whether they include only the simulation of the gastric phase or also the small intestine phase), in the pH value and chemical composition of the synthetic digestion fluids used, in the ratios between the solid substance (sample) and the solution, in the number and type of added enzymes and food, and in the duration of the entire test (Juhasz et al., 2009). These differences prevent comparison of results. That’s why a Bioaccessibility Research Group of Europe (BARGE) developed a Unified BARGE Method (UBM) for determination of oral bioaccessibility. With the development of UBM, they unified the procedures for determining oral bioaccessibility, introduced a method with adequate accuracy and precision of analyses and adequate correlation with in vivo methods (Denys et al., 2012; Wragg et al., 2011).

The UBM was firstly developed and validated for assessing bioaccessibility of As, Cd, and Pb in soil (Denys et al., 2012; Wragg et al., 2011) but during the following years its use was expanded to the other PTEs (e.g. Barsby et al., 2012; Cruz et al., 2015; Qin et al., 2016; Shi et al., 2023; Vasiluk et al., 2023), other media, like household dust (e.g. Marinho-Reis et al., 2020; Zupančič et al., 2021) and rice (Ma et al., 2024), and also to other anthropogenic contaminants, like microplastics (López-Vázquez et al., 2022) and polycyclic aromatic hydrocarbons (Armada et al., 2023).

The aims of our study were the following: (1) to analyse and compare the oral bioaccessibility of PTEs in four different solid media (soil, attic dust, street dust, household dust) that people are most often exposed to in urban environments; (2) to analyse oral bioaccessibility of a wide range of PTEs, including some that are not often studied (e.g., Ce, La, Li, Sn) but might pose a human health hazard, today or in the future due to their increasing usage; and (3) to test the feasibility of usage of scanning electron microscope techniques in analyses of solid residuals of gastric and gastro-intestinal phases of UBM. The foundations for presented bioaccessibility study were extensive geochemical studies of Maribor urban area done in the last years (Gaberšek & Gosar, 2018, 2021a, 2021b; Gaberšek et al., 2020). Bioaccessibility of ten PTEs in attic dust has been partially already published in Gaberšek et al. (2022), while other data in the present paper has not yet been presented.

## Materials and methods

### Study area

**Fig. 1:**
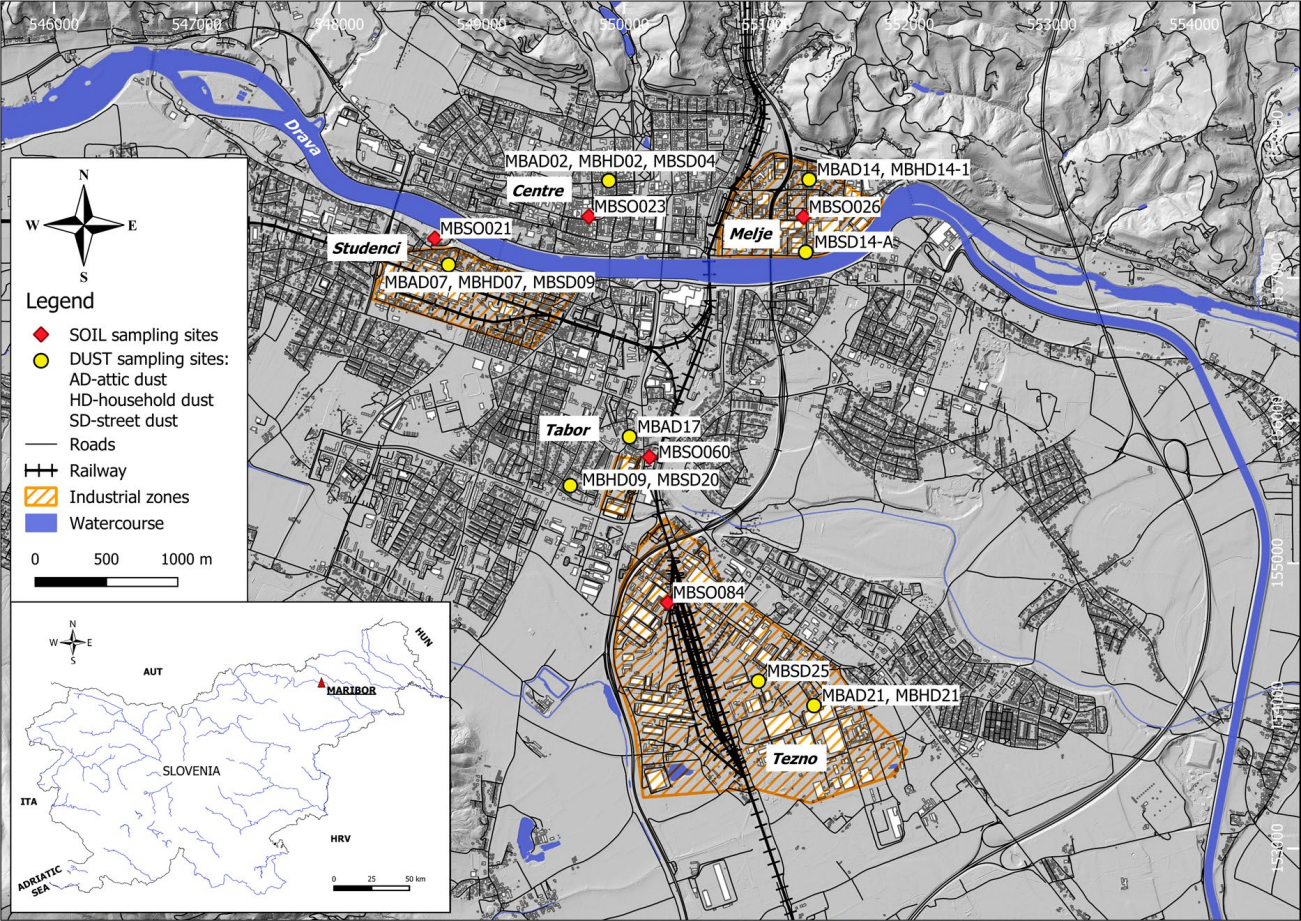
Geographical location of study area and sampling sites on schematic map of Maribor

As a test site for presented study of oral bioaccessibility of PTEs in various media, we selected the town of Maribor, Slovenia (46°32′ N, 15°39′ E, 275 m above sea level (Fig. 1). It is the second largest Slovenian town with 95,000 inhabitants and it is an industrial and economic centre located at an intersection of important international routes. Industrial facilities are mainly concentrated in the town's two industrial zones, called Melje and Tezno. One of the most important industrial sectors is the metal industry, with foundry and a metal furniture factory located in Melje, and automotive and metal processing factories in the Tezno area.

The town of Maribor developed on the Drava River alluvial plain, which is represented by Quaternary glacio-fluvial and fluvial sediments, consisting mainly of metamorphic and igneous rock fragments (Mencin Gale et al., 2019; Mioč & Žnidarčič, 1989; Šoster et al., 2017; Trajanova, 2002; Žlebnik, 1982). The surroundings of the town are dominated by the hilly area of Slovenske Gorice, consisting of Miocene sedimentary rocks (mainly siliciclastic rocks), and by the Pohorje and Kozjak Mountains, consisting of a variety of metamorphic and igneous rocks (Hinterlechner Ravnik, 1971; Mioč, 1978; Mioč & Žnidarčič, 1989; Zupančič, 1994). Additional information about the historical industrial development of Maribor, the geological setting and soil types of the study area can be found in Gaberšek and Gosar (2018, 2021b).

Extensive geochemical studies of Maribor urban area were done in the last years (Gaberšek & Gosar, 2018, 2021a, 2021b; Gaberšek et al., 2020, 2022). The first systematic geochemical survey of urban soil of Maribor showed a relatively clear distinction between naturally and anthropogenically distributed elements, with the median levels of Cu, Pb and Zn significantly higher than the corresponding Slovenian median levels defined by Gosar et al. (2019) and Pučko et al. (2024). Enrichments of these three PTEs were typically found in industrialised zones and in the old town centre (Gaberšek & Gosar, 2018). The town of Maribor was used as a test site in study of Gaberšek and Gosar (2021b) that aimed to introduce a multi-media, multi-analytical and multi-elemental holistic approach to geochemical studies of inorganic PM in urban areas. The chemical composition and individual particulate characteristics of street, attic and household dust were determined and compared with the characteristics of airborne PM, and PM deposited in snow (Gaberšek & Gosar, 2021a), together with the chemical composition of the soil. It was discovered that the mineralogical and chemical composition and the individual solid particle characteristics of the studied media differ considerably. The highest levels of PTEs in all media, except household dust, are typical for industrial areas. Street dust primarily reflects the influence of winter road maintenance and industrial activities, while characteristics of household dust are predominantly influenced by indoor activities and properties of apartments. The most recent sources of PTE-bearing particles can be identified by the characterisation of airborne PM and PM deposited in snow. Several industrial sources and the fate of some particle types in the environment have been determined based on the findings of the SEM/EDS analyses by this research.

### Sampling and sample preparation

Sampling of **soil** (sample code: MBSO), **attic dust** (sample code: MBAD), **household dust** (sample code: MBHD) and **street dust** (sample code: MBSD) was done in the frame of geochemical research of the Maribor urban area. A total of 118 samples of soil was collected (Gaberšek & Gosar, 2018), 19 samples of attic dust (Gaberšek et al., 2022) and 27 samples of household dust and 33 samples of street dust (Gaberšek & Gosar, 2021b). Oral bioaccessibility of selected PTEs was analysed in 5 samples of each media (a total of 20 samples). Selection of samples was based on results of preliminary geochemical analyses and their spatial distribution as we wanted to cover different districts of the town with different land use (industrial and residential). Samples chosen for UBM analysis were collected in the following five town’s district (Fig. 1): old town centre (MBSO023, MBAD02, MBHD02, MBSD04), Studenci industrial zone (MBSO021, MBAD07, MBHD07 MBSD09), Melje industrial zone (MBSO026, MBAD14, MBHD14-1, MBSD14-A), newer residential area close to the Tezno industrial zone called Tabor (MBSO060, MBAD17, MBHD09, MBSD20), and Tezno industrial zone (MBSO084, MBAD21, MBHD21, MBSD25).

The protocol of soil and dust sampling mostly followed the procedures described in the EuroGeoSurveys Urban Geochemical Mapping Manual (Demetriades & Birke, 2015). Soil samples were collected from the top 10 cm of soil using a stainless-steel trowel and packed in polyethylene bags. Sampling sites were located mostly in parks, on green spaces and lawns. Samples were oven dried at 35 °C to prevent the loss of volatile elements. Dried soil aggregates were then gently disaggregated in a mortar with a ceramic pestle, homogenised and passed through a 2 mm nylon sieve. Sieved samples were pulverised in an agate ball mill to obtain analytical grain size of < 0.075 mm (Gaberšek & Gosar, 2018). Although the recommended size fraction of UBM protocol is < 0.250 mm, we decided to use smaller size fraction as smaller particles tend to adhere more effectively to human hands, and thus can be involuntarily ingested by humans (Siciliano et al., 2009). Additionally, Juhasz et al. (2011) stated that using < 250 μm size fraction has the potential to underestimate Pb exposure due to the preferential adhesion of smaller particles with elevated Pb concentrations to hands. Attic dust was sampled by brushing the dust off the surface of wooden building elements in uninhabited and uninsulated attics, in buildings which were mostly older than 80 years old (Gaberšek et al., 2022). Street dust samples were collected by brushing the paved areas along the curbs with plastic brushes. Each sample consisted of several subsamples. Household dust was sampled by collecting vacuum cleaner bags that had been used for one to three months. Participating residents also completed a questionnaire on their lifestyle, habits and the characteristics of the dwellings that could influence the chemical composition of the household dust there (Gaberšek & Gosar, 2021b). All dust samples were oven-dried at 35 °C and sieved at < 0.063 mm using nylon sieves. Sample preparation was done at Geological Survey of Slovenia.

### Determination of total levels of PTEs and their oral bioaccessibility–unified BARGE method

Total levels and oral bioaccessibility of the following 13 elements were analysed: As, Cd, Ce, Cr, Cu, Hg, La, Li, Ni, Pb, Sb, Sn and Zn. For determination of total levels, a 0.25 g of each sample was digested in PFA beakers with a mixture of HF-HNO_3_-HClO_4_ acids and total levels were determined by ICP-MS. Levels of Hg were determined by Direct Mercury Analysis System DMA-80.

Oral bioaccessibility was determined using the in vitro Unified BARGE Method (UBM). UBM simulate the physico-chemical conditions in the human mouth, stomach, and small intestine (e.g. pH, temperature, presence of enzymes, peristalsis, resident time) using the following synthetic analogue digestive fluids: saliva (pH = 6.5 ± 0.5), gastric (pH = 1.1 ± 0.1) and duodenal (pH = 7.4 ± 0.2) fluids, and bile (pH = 8.0 ± 0.2). Detailed compositions of digestive fluids are given in Wragg et al., (2009, 2011). The following description of the method is partially summarised after Wragg et al., (2009, 2011) and it was described in Gaberšek & Gosar (2022). The UBM procedure runs in two parallel phases (gastric (G) and gastro-intestinal (GI) phase) which results in two supernatants of each sample at the end of the procedure. A 0.6 g of sample is used for each phase. The UBM method consists of three stages. In the first stage, sample is mixed with 9.0 mL of saliva and shaken for 10 s. Then 13.5 mL of gastric fluid is added and the pH is checked. If pH is not 1.2, adjustment with 1 M NaOH and/or 37% HCl is performed. This procedure is repeated until pH stabilizes at 1.2 ± 0.05. The reaction tubes were then placed in an end-over-end rotator which is submerged in a water bath heated at 37 °C. After one hour of agitation, the pH is checked. The whole procedure should be repeated if the pH was higher than 1.5. The gastric phase samples are then centrifuged for 15 min at 4500 g. Supernatants of gastric phase were collected by pipetting and acidified with 500 µL of HNO_3_ (67%). On the other hand, the gastro-intestinal phase continues with adding 27 mL of duodenal fluid and 9 mL of bile fluid and adjusting the pH to 6.3 ± 0.5 with NaOH or HCl. Tubes are then again placed into end-over-end rotator submerged in a water bath (37 °C). After four hours of agitation, the pH is noted and samples are centrifuged for 15 min at 4500 g*.* Supernatants of gastro-intestinal phase were collected and acidified with 1.0 mL of HNO_3_ (67%). The levels of 13 PTEs in both phases were measured with ICP-MS. Bioaccessible fractions (BAFs) of each PTE in both phases were calculated using Eq. (1).

1 $${\text{BAF}}\left( \% \right) = \frac{{{\text{Bioaccessible level of PTE }}\left( {\frac{{{\text{mg}}}}{{{\text{kg}}}}} \right)}}{{{\text{Total level of PTE }}\left( {\frac{{{\text{mg}}}}{{{\text{kg}}}}} \right)}} \times 100\;(\% )$$

Determination of total levels of PTEs and their oral bioaccessibility were performed at the British Geological Survey (BGS).

### Quality control

The quality control of chemical analyses was ensured in several ways. Samples of four Certified Reference Materials (CRM: 2711a, BCR-2, BGS-102, NIST 2584) and replicates of two dust samples were included randomly into the sample batch to estimate accuracy and precision, respectively, of the chemical analysis for determination of total levels of PTEs. Accuracy was estimated by calculating percentage recovery (PR) and precision by calculating relative percent difference (RPD). The accuracy of Hg and Sn could not be determined. The mean PRs were between 87% (Cr) and 118% (Cd), and mean RPDs were between 1.3% (Ni) and 8.5 (Cd). The quality of analysis of total levels were thus satisfactory for all elements.

The accuracy of the UBM extraction protocol was estimated by analysing the BGS guidance material 102 (replicated 5 times), which provides reliable guidance values for 9 of the 13 analysed PTEs (exceptions are Hg, Li, Sb, Sn) in gastric phase (Hamilton et al., 2015). The accuracy, expressed as relative error (RE), was < 6% for all 9 PTEs, for which guidance values are available in the literature (Hamilton et al., 2015). The exceptions are As (RE = 14.7%) and Ni (RE = 7.3%). The replicate of samples MBSO084, MBAD21, MBHD21, and MBSD25 were added to sample batch in both phases to establish the precision of the analyses. The precision, expressed as mean RPD, was between 1.6% (Zn) and 13.3% (Hg) in the gastric phase, and < 20% for all PTE, except for Sn (RPD = 21.7%), Li (RPD = 22.2%), La (RPD = 25.8%), Ce (RPD = 29.0%), Pb (RPD = 34.5%), and Cd (RPD = 36.9%) in the gastro-intestinal phase. Additionally, a blank sample (containing only synthetic digestive fluids) was also analysed. As Zn is a significant component of the extraction fluids, 8.7 mg/kg and 13.9 mg/kg of Zn was detected in gastric and gastro-intestinal phase, respectively, in blank sample. These Zn levels in blank sample were subtracted from levels of individual sample. The overall quality of UBM analyses was satisfactory for all elements. Only the precision of Pb and Cd in gastro-intestinal phase was somewhat lower (RPD between 30 and 40%) so these two elements should be interpreted with prudence.

Additionally, gastric bioaccessible levels of Cd in MBHD21 and MBSD09, of Cu in MBHD09, and of Zn in MBHD09 were higher than total levels. These results were excluded from further discussion. The following bioaccessible levels were below detection limits (DL): levels of Hg in gastric phase of MBHD02 (DL < 0.001 mg/kg), Sn in gastric phase of MBHD02, MBHD07, and MBHD14-1 (DL < 0.008 mg/kg), and Sn in gastro-intestinal phase of MBSO021, MBSO026, MBSO060, MBSD04, MBSD09, MBSD20, and MBSD25 (DL < 0.020 mg/kg).

An important quality control aspect of UBM is the control of pH of digestive fluids. The final gastric phase pH for samples MBSO060 (soil), MBAD21 (attic dust), MDHD02, MBHD07, MBHD14-1 (all household dust), and all five samples of street dust were over the tolerance specified in the method (final pH of < 1.5) and repeat extraction duplicated this observation. These observations are likely due to the nature of the samples as the high Ca content may buffer any pH changes during the extraction procedure. Additionally, the quality control of gastro-intestinal phase is particularly challenging because significant changes in physico-chemical conditions occur after the transition from the stomach to the small intestine (Wragg et al., 2011), and resulting processes are difficult to satisfactorily mimic with in vitro assays.

### Analysis of solid residual with SEM/EDS

Selected solid residual of gastric and gastro-intestinal phases were analysed with scanning electron microscopy coupled with energy-dispersive spectroscopy (SEM/EDS) to characterise the micromorphological and chemical changes of individual particles occurring during the UBM and to determine the usability of this method in bioaccessibility studies. The samples of solid residual were placed on double-sided carbon tape with a surface area of about 25 mm^2^ and carbon-coated to obtain the conductivity of the samples. The analysis was performed with a JEOL JSM 6490LV SEM coupled with an Oxford INCA EDS system consisting of an Oxford INCA PentaFET3 Si(Li) detector and INCA Energy 350 processing software with the following settings: high vacuum, accelerating voltage of 20 kV, spot size 50, working distance of 10 mm, EDS acquisition time of 60 s, backscattered electrons mode (BSE).

## Results and discussion

### Bioaccessibility of PTEs in different media

Table 1 comprises minimum, mean, and maximum total levels of analysed 13 PTEs, and data on their minimum, mean, and maximum bioaccessible levels in gastric and gastro-intestinal phases as well as calculated bioaccessible fractions (BAFs). The data for individual samples are provided as Supplementary Material (SM1). Determined bioaccessible fractions (BAFs) differ strongly between individual PTEs, individual samples of the same medium, different media, and the gastric and gastro-intestinal phases of the UBM. Among the most bioaccessible PTEs in the gastric phase in all four media are Cd, Cu, Pb and Zn, with the exception of Pb in household dust. Even if we calculate the mean BAFs of PTEs for the whole data set (not separated by media), the most bioaccessible are the same four PTEs. The mean gastric BAF of Cd is 53%, of Zn 48%, of Pb 34% and of Cu 28%. Mean BAFs of As, Ni and La are between 10 and 20%, while Ce, Cr, Sb, and Li have BAFs between 5 and 10%. Mercury and Sn are the least bioaccessible PTEs in gastric phase, with mean gastric BAFs of 3.8% and 2.2%, respectively. In the gastro-intestinal phase, which is characterized by a close-to-neutral pH and the possibility of recrystallisation of PTEs that has been dissolved in previous (gastric) phase, the most bioaccessible PTEs are partly other than in the gastric phase. The highest mean BAF has Cu (35%, which is slightly more than in the gastric phase), followed by Cd (23%), Ni and As (both 15%). BAFs between 5 and 10% have Sb, Zn, Li, and below 5% Hg (4.3%), Cr (3.5%), Pb (2.9%), Sn (1.1%), La (1.0), and Ce (0.7%). During the transition from stomach to the small intestine, the mobility and consequently the mean BAFs of most PTEs decreases, most strongly for Cd (by 30.6%), Pb (by 31%) and Zn (by 39.8%). The exceptions are Cu, Hg and Sb, which mean BAFs slightly increase. The bioaccessibility of Li remains unchanged.

**Table 1:** Minimum (min), mean, and maximum (max) total levels of analysed 13 PTEs and their bioaccessible levels in gastric and gastro-intestinal phases (given in mg/kg), and calculated bioaccessible fractions (BAFs, given in %) in soil (SO), attic dust (AD), household dust (HD, and street dust (SD)

PTE	Medium		Total level (mg/kg)	Gastric phase		Gastro-intestinal phase	
				(mg/kg)	(%)	(mg/kg)	(%)
As	SO	Min–Max	8.8–24.4	0.85–6.82	6.8–27.9	0.73–3.99	5.84–16.3
		Mean	14.4	2.32	14.0	1.65	10.7
	AD	Min–Max	15.5–40.4	2.54–13.7^1^	16.4–42.4	1.74–13.5^1^	11.2–33.3
		Mean	27.3	9.03^1^	31.5	6.66^1^	22.4
	HD	Min–Max	2.99–8.97	0.23–0.40	2.88–7.61	0.59–3.03	13.9–33.8
		Mean	6.43	0.27	4.88	1.57	22.5
	SD	Min–Max	5.30–7.45	0.67–3.24	9.10–58.0	0.21–0.44	3.12–6.08
		Mean	6.20	1.74	29.9	0.28	4.59
Cd	SO	Min–Max	0.58–2.03	0.37–1.66	54.3–81.8	0.14–0.46	10.5–24.7
		Mean	1.22	0.79	62.2	0.23	19.8
	AD	Min–Max	2.17–7.92	1.23–5.49^1^	56.7–69.4	0.41–3.14^1^	18.9–42.1
		Mean	4.19	2.81^1^	65.2	1.42^1^	31.9
	HD	Min–Max	0.71–5.16	0.28–1.18	5.39–100	0.12–1.96	10.1–37.9
		Mean	1.93	0.79	59.7	0.62	27.5
	SD	Min–Max	0.97–7.45	0.55–1.0	13.5–37.6	0.10–0.64	7.24–17.6
		Mean	3.88	0.812	21.1	0.40	11.6
Ce	SO	Min–Max	45.0–60.7	3.11–6.33	6.91–10.4	0.11–0.86	0.23–1.63
		Mean	52.0	4.21	7.98	0.52	0.97
	AD	Min–Max	37.4–51.4	2.53–12.5	6.76–24.2	0.20–0.70	0.52–1.62
		Mean	44.2	6.85	15.0	0.36	0.82
	HD	Min–Max	25.0–85.8	0.11–0.33	0.26–0.70	0.10–1.12	0.36–2.35
		Mean	44.7	0.19	0.45	0.38	0.85
	SD	Min–Max	40.2–45.3	3.47–11.9	8.11–27.4	0.07–0.14	0.16–0.34
		Mean	42.3	6.29	14.8	0.10	0.23
Cr	SO	Min–Max	74.1–122	1.29–6.96	1.68–6.84	0.15–0.58	0.20–0.62
		Mean	91.6	3.60	3.60	0.38	0.41
	AD	Min–Max	138–322	9.77–13.3^1^	3.67–9.45	1.82–3.29^1^	0.66–2.19
		Mean	182	12.0^1^	7.27	2.52^1^	1.54
	HD	Min–Max	80.8–181	0.44–3.16	0.31–2.84	3.82–63.2	3.62–34.9
		Mean	117	1.12	1.04	17.9	12.1
	SD	Min–Max	113–2041	9.59–82.6	0.73–61.4	0.45–0.71	0.04–0.16
		Mean	566	28.0	18.4	0.32	0.11
Cu	SO	Min–Max	60.1–689	14.1–355	23.5–54.9	15.3–365	25.4–53.0
		Mean	308	147	39.5	142	38.1
	AD	Min–Max	147–2216	17.3–939^1^	8.38–46.4	38.6–1005^1^	18.7–46.5
		Mean	583	233^1^	35.3	251^1^	38.4
	HD	Min–Max	89.5–204	2.57–18.7	1.70–20.8	40.4–76.9	37.8–45.1
		Mean	149	10.1	8.20	62.0	42.3
	SD	Min–Max	122–6842	30.1–55.2	0.64–45.3	29.4–807	11.8–25.5
		Mean	1533	46.0	26.0	193	19.8
Hg	SO	Min–Max	0.14–1.1	0.003–0.008	0.55–2.35	0.004–0.008	0.61–3.02
		Mean	0.55	0.005	1.54	0.006	1.79
	AD	Min–Max	0.18–0.79	0.006–0.079^1^	3.31–10.0	0.011–0.086^1^	4.89–11.0
		Mean	0.39	0.029^1^	6.94	0.032^1^	7.63
	HD	Min–Max	0.24–0.77	0.001–0.002	0.18–0.68	0.007–0.016	1.40–4.42
		Mean	0.52	0.001	0.38	0.011	2.33
	SD	Min–Max	0.04–4.7	0.003–0.007	0.07–10.1	0.004–0.005	0.11–12.6
		Mean	1.02	0.005	5.71	0.005	5.28
La	SO	Min–Max	22.7–31.3	2.14–4.33	9.45–14.5	0.08–1.04	0.34–3.50
		Mean	27.3	3.25	11.7	0.46	1.60
	AD	Min–Max	20.6–27.5	1.81–7.59	8.81–27.6	0.13–0.38	0.64–1.71
		Mean	23.5	4.14	17.0	0.21	0.90
	HD	Min–Max	12.8–48.9	0.11–0.29	0.33–1.14	0.06–0.85	0.40–3.34
		Mean	24.4	0.17	0.81	0.27	1.14
	SD	Min–Max	19.8–23.2	2.34–7.70	10.1–34.9	0.06–0.09	0.28–0.47
		Mean	21.6	4.17	19.3	0.08	0.36
Li	SO	Min–Max	25.3–38.5	0.25–1.67	0.75–6.59	0.23–0.70	0.69–2.77
		Mean	33.1	0.59	2.05	0.39	1.27
	AD	Min–Max	27.9–37.9	2.66–6.90	9.53–18.2	3.09–5.69	10.9–15.1
		Mean	31.2	4.21	13.2	4.11	13.0
	HD	Min–Max	9.47–19.7	0.27–0.67	1.68–4.49	0.83–2.11	4.20–15.4
		Mean	14.2	0.47	3.45	1.43	10.7
	SD	Min–Max	13.6–19.0	0.99–2.06	5.63–11.6	0.56–0.61	1.89–3.20
		Mean	16.6	1.41	8.53	0.39	2.32
Ni	SO	Min–Max	34.7–73.4	2.37–14.8	6.30–20.6	1.73–12.1	4.38–16.4
		Mean	44.9	6.14	12.3	4.30	8.26
	AD	Min–Max	50.4–123	9.11–21.0^1^	14.2–29.4	8.23–20.2^1^	16.3–26.0
		Mean	74.3	15.4^1^	21.4	14.2^1^	19.3
	HD	Min–Max	37.8–85.9	1.67–9.34	2.35–22.4	10.9–30.0	20.7–34.9
		Mean	55.4	4.54	10.2	16.4	29.7
	SD	Min–Max	44.0–243	12.0–27.8	4.95–56.7	1.58–8.75	2.42–5.33
		Mean	117	16.9	27.2	4.22	4.15
Pb	SO	Min–Max	85.8–437	49.5–255	39.4–61.6	3.84–18.9	1.63–9.28
		Mean	233	117	51.6	10.9	5.46
	AD	Min–Max	340–1024	18.2–343^1^	5.35–68.4	2.98–19.9^1^	0.88–4.52
		Mean	502	210^1^	44.2	10.6^1^	2.14
	HD	Min–Max	53.1–237	1.13–4.93	0.50–4.77	1.47–7.59	1.28–7.34
		Mean	127	2.25	2.26	3.18	2.90
	SD	Min–Max	116–450	24.2–116	5.37–99.9	1.14–4.48	0.48–1.90
		Mean	235	59.8	37.3	2.18	1.01
Sb	SO	Min–Max	1.53–9.62	0.04–0.27	2.33–4.77	0.08–0.43	3.99–6.95
		Mean	4.58	0.14	3.19	0.22	5.25
	AD	Min–Max	8.13–20.0	0.73–3.91^1^	6.81–19.5	0.93–4.17^1^	9.60–20.8
		Mean	12.7	1.70^1^	12.4	1.86^1^	13.5
	HD	Min–Max	3.68–11.5	0.06–0.28	0.55–5.18	0.40–1.51	6.56–25.0
		Mean	6.66	0.14	2.68	0.89	14.8
	SD	Min–Max	3.87–17.7	0.25–1.13	1.84–29.2	0.09–0.39	1.54–2.87
		Mean	10.2	0.70	10.8	0.21	2.08
Sn	SO	Min–Max	7.51–41.4	0.019–0.434	0.26–1.48	< 0.020–0.092	/ -0.312
		Mean	20.0	0.168	0.78	0.061 (N = 2)	0.31 (N = 2)
	AD	Min–Max	19.8–75.6	0.076–2.17^1^	0.39–3.61	0.027–0.282^1^	0.136–0.667
		Mean	34.3	0.868^1^	2.35	0.127^1^	0.343
	HD	Min–Max	7.18–27.7	< 0.008–0.061	/ -0.52	0.126–0.802	1.08–3.29
		Mean	15.5	0.035 (N = 2)	0.32 (N = 2)	0.357	2.38
	SD	Min–Max	17.7–68.6	0.255–2.19	0.37–7.87	< 0.020–0.115	/ -0.168
		Mean	40.6	1.10	4.10	/	/
Zn	SO	Min–Max	236–1162	58.1–711	20.8–70.7	1.24–48.3	0.53–5.69
		Mean	706	355	43.0	20.9	2.36
	AD	Min–Max	929–6304	558–3689^1^	51.4–67.3	48.2–1097^1^	5.19–17.4
		Mean	2298	1338^1^	58.3	339^1^	12.1
	HD	Min–Max	567–1497	248–456	27.4–80.4	66.7–166	8.27–15.0
		Mean	851	360	45.0	95.8	11.5
	SD	Min–Max	1042–4395	360–1042	8.19–100	39.8–267	2.80–7.50
		Mean	1855	571	44.1	104	5.52

^1^Data has been partially used in Gaberšek et al. (2022)

**Fig. 2:**
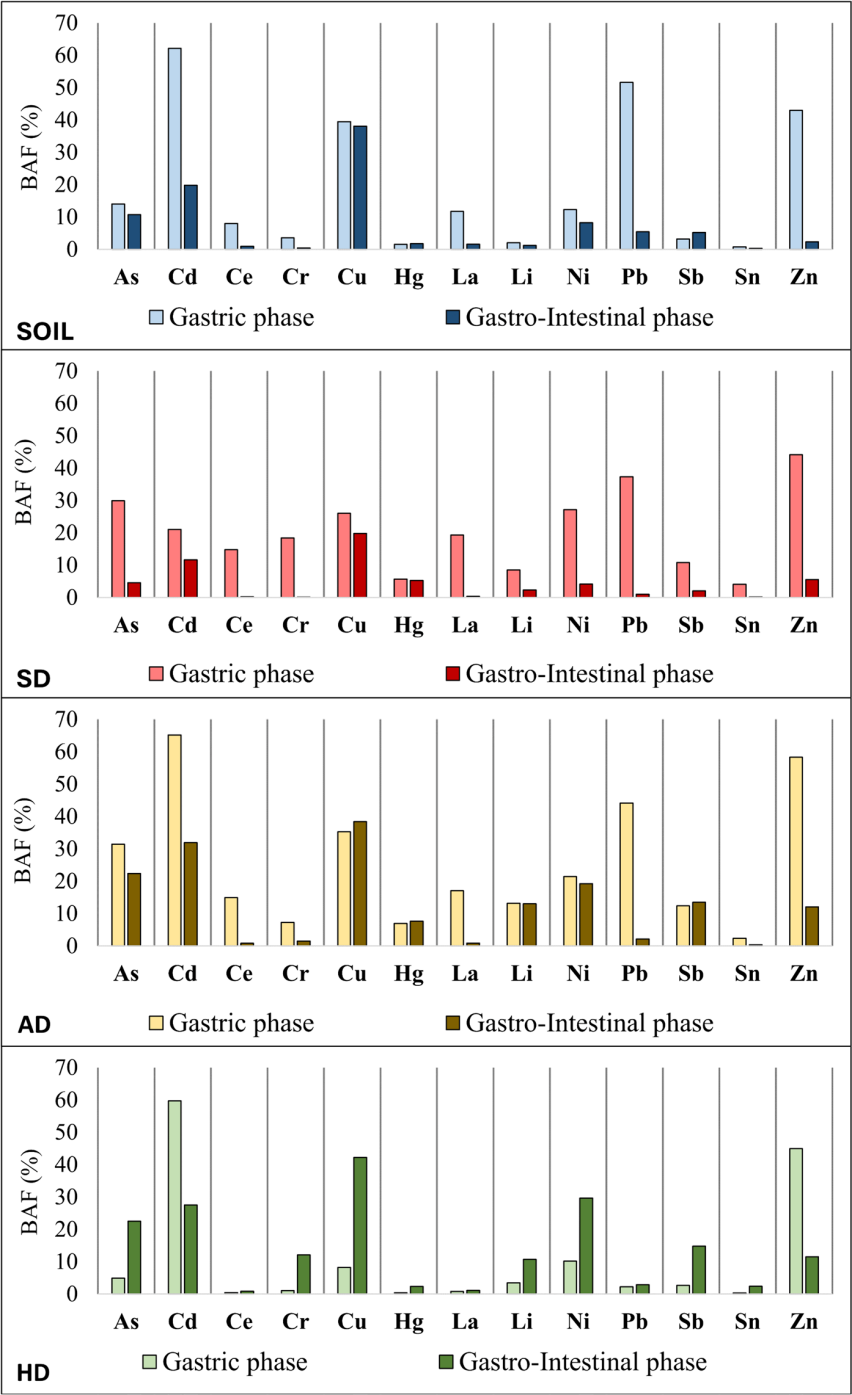
Mean bioaccessible fractions (BAF) of all 13 PTEs in soil, street dust (SD), attic dust (AD), and household dust (HD) (data for AD has been partially published in Gaberšek et al. (2022))

A more detailed description of bioaccessibility by individual medium is given below. Most PTEs in **soil** samples have much higher BAFs in gastric phase than in the gastro-intestinal phase (Fig. 2). The only exceptions are Hg and Sb, which have a slightly lower BAFs in the gastric phase. Cadmium, Cu, Pb and Zn have the highest mean gastric BAFs. The total levels of these four PTEs in the selected five soil samples are much higher than the mean and median levels in the soil in the entire area of Maribor (Gaberšek & Gosar, 2018), that’s why we assume that they at least partly originate from anthropogenic sources. The mean gastric BAF of Cd is 62% (range of 54–82%), of Cu 40% (range of 24–55%), of Pb 52% (range of 39–62%) and of Zn 43% (range of 21–71%). The bioaccessibility of Cd, Pb and Zn is much lower in the gastro-intestinal phase, the differences between the two phases ranging from 41% (Zn) to 46% (Pb). In the case of Cu, the difference between the mean BAFs in the gastric (40%) and gastro-intestinal phases (38%) is minimal. The least bioaccessible elements in soil (mean values are given in the brackets) are in the gastric phase Sn (0.78%), Hg (1.5%), and Li (2.1%), and in the gastro-intestinal phase Ce (0.97%), Cr (0.41%), Hg (1.8%), La (1.6%), Li (1.3%) and Sn (3 values < DL).

There are numerous studies of bioaccessibility of PTEs in soil in which UBM was used (e.g. Barsby et al., 2012; Qin et al., 2016; Waterlot et al., 2017; Zhu et al., 2019; Fernandez-Landero et al., 2021; Cocerva et al., 2024). Although results of these studies somewhat differ, there are some common observations and similarities with the presented study. Most PTEs have higher BAFs in gastric phase than in gastrointestinal phase. Furthermore, PTEs with the highest BAFs in gastric phase are usually Cd, Cu, Pb, and Zn, while the most bioaccessible PTEs in gastrointestinal phase are various. Additionally, relatively high correlation between total levels and their bioaccessible levels are usually determined. For example, the mean gastric BAFs of Cd in Northern Ireland soil was 49%, of Pb 33%, of Cu 31%, and of Zn 22% (Barsby et al., 2012), which is slightly lower than in Maribor soil. The BAFs of As, Cr and Ni are almost the same in both cases. The BAFs of gastro-intestinal phase are also very similar between the two studies. The exceptions are Cu, which mean BAF is 10% higher in Maribor samples, and Pb and Zn, which are slightly more bioaccessible in soil of Northern Ireland. Barsby et al. (2012) highlighted the strong influence of differences in soil parent materials on oral bioaccessibility of PTEs as some soil showed elevated pseudo-total PTEs levels while the measured BAFs were relatively low, and vice versa. Another study performed by Waterlot et al. (2017) got similar results, with mean gastric BAFs of Cd to be 65%, Pb 56% and Zn 59%. The gastro-intestinal BAFs of all three PTEs were 31%, which is much higher than in the Maribor’s soil. They concluded that the cation exchange capacity and phosphorous contents in soils are the two main parameters controlling the bioaccessibility of Cd, Pb and Zn in the gastric phase and that bioaccessible metals in soil were present mainly in a soluble form, weakly bound to the organic matter and associated with carbonates and Fe and Mn oxides/hydroxides (Waterlot et al., 2017). According to literature data, large ranges in BAFs of the same PTEs in different samples is also very typical. Zhu et al. (2019) found that gastric BAFs of As varied between 0.79 and 18%, Cd between 5.8 and 92% and Pb between 0.1 and 51%. Large ranges were also found by Finžgar et al. (2014) in soil samples from the area of former Pb–Zn mine of Mežica, Slovenia. The BAFs of Pb in the gastric phase were 45–68% and in the gastro-intestinal phase 4.3–10.8%. As Zn is present in mostly inert chemical/mineral phases, its bioaccessibility (3.7–5.4% in gastric phase, and 0.44–0.92% in gastro-intestinal phase) was much lower than bioaccessibility of Pb in Mežica soil and also much lower than bioaccessibility of Zn in Maribor soil. Besides pH and chemical speciation of PTEs, the other factors controlling bioaccessibility of PTEs in soil are particle size (a general trend of higher bioaccessibility in finer size fraction was found (Li et al., 2021)), and organic matter, clay, and reactive iron contents (Walraven et al., 2015). Good et al. (2024) determined that Pb bioaccessibility is lower in soil with higher organic matter content. They highlighted also the importance of distance from PTEs sources as samples closer to former Pb smelter sites had a somewhat higher average BAFs (Good et al., 2024). Bioaccessibility of PTEs also tends to be much lower when their origin is geogenic compared with anthropogenic contamination (Billmann, et al., 2023) which is often the case in old industrial urban areas, such as Maribor.

The most bioaccessible PTEs in gastric phase of **attic dust** (Fig. 2) are the same as in soil, as follows: Cd (mean of 65%; range of 57–67%), Zn (mean of 58%; range of 51–57%), Pb (mean of 44%; range of 5.4–68%) and Cu (mean of 35%; range of 8.4–46%). They are followed by As (mean of 32%; range of 16–42%), which has much higher BAFs in attic dust than in soil. Tin (mean of 2.4%), Hg (mean of 6.9%) and Cr (mean of 7.3%) have the lowest mean gastric BAFs. In the gastro-intestinal phase, Cu (mean of 38%; range of 19–47%), Cd (mean of 32%; range of 19–42%), and As (mean of 22%; range of 11–33%) are the most bioaccessible, and Sn (mean of 0.34%), Ce (mean of 0.82%), La (mean of 0.90%), and Cr (mean of 1.5%) are the least. Nine out of 13 PTEs have a higher mean BAF in the gastric phase than in the gastro-intestinal phase. Copper, Hg and Sb have slightly higher BAFs in the second phase, while Li values are practically the same in both phases. To the best of our knowledge, the oral bioaccessibility of PTEs in attic dust was studied only twice until now and only once UBM was used. Bačeva-Andonovska et al. (2015) simulated gastric conditions by using 0.1 mol/l HCl acid. The results showed relatively high BAFs of Cr, Cu, Mn, Ni, Pb, and Zn in samples taken close to the metallurgical activities, which the authors explained by the small size fraction and high reactivity of the particles of attic dust. The second example of bioaccessibility study of attic dust is the present study which has been partially published in Gaberšek et al. (2022).

All PTEs in **street dust** are more bioaccessible in the gastric than in the gastro-intestinal phase (Fig. 2). The highest mean gastric BAFs have Zn (mean of 44%; range of 8.4–100%), Pb (mean of 37%; range of 5.4–100%), As (mean of 30%; range of 9.1–58%), Ni (mean of 27%; range of 5.0–57%) and Cu (mean of 26%; range of 0.64–45%) and the lowest Sn (mean of 4.1%) and Hg (mean of 5.7%). Bioaccessibility in the gastro-intestinal phase is significantly lower, the highest mean BAFs have Cu (mean of 20%; range of 12–53%) and Cd (mean of 12%; range of 7.2–18%). Research of bioaccessibility of PTEs in street dust is also rare, despite the fact that street dust has a high ability for accumulating PTE-bearing solid particles of anthropogenic origin on the one hand, and the possibility of their resuspension into the atmosphere and thus presenting a health hazard on the other hand. Pelfrêne and Douay (2018) determined the bioaccessibility of Cd and Pb in dust deposited on asphalt pavements from an area characterised by a former Pb smelter. They analysed BAFs in five particle size fractions. They found that both elements have the highest BAFs in the smallest fraction (< 5 µm), as is also typical for BAFs in other media. In the fraction most comparable to the size used in our study (5–50 µm), the gastric BAF of Cd is 41 ± 16.8% and Pb 61 ± 16.8%. Gastro-intestinal BAF is much smaller; it is 17.6 ± 9.1% for Cd and 11.2 ± 7.7% for Pb.

The bioaccessibility of PTEs in **household dust** differs strongly from the rest of the media, as 11 out of 13 studied PTEs have higher mean BAFs in the gastro-intestinal phase than in the gastric phase (Fig. 2). The opposite is true only for Cd and Zn, which also have the highest mean gastric BAFs (60% and 45%, respectively). The mean gastric BAFs of other PTEs are below 10%, with Ce, Hg, La and Sn having the lowest. The highest mean BAFs in the gastro-intestinal phase were determined for Cu (mean of 42%; range of 38–45%), Ni (mean of 30%; range of 21–35%), Cd (mean of 28%; range of 10–38%) and As (mean of 23%; range of 14–34), and the lowest for Ce (mean of 0.85%) and La (mean of 1.1%). Comparison of literature BAFs between the media indicate that BAFs in household dust may be higher than in other media. For example, mean BAFs of Cd, Cu, Pb, and Zn in gastric phase in household dust from the town of Idrija, Slovenia (Zupančič et al., 2021) are 80%, 35%, 82%, and 77%, respectively. Similarly high gastric mean BAFs of Cd (81%), Pb (60%), Zn (84%) and also relatively high BAFs of Ni (40%), Cu (30%), Cr (22%), and Sb (13%) in household dust were determined also by Marinho-Reis et al. (2020). Interestingly, this is not the case in our study as mean gastric BAFs of PTEs in household dust are much lower than in other media, especially in case of Cu (mean gastric BAF of 8.2%) and Pb (mean gastric BAF of 2.3%). It seems that several PTEs occur in more inert chemical/mineral phases in household dust from Maribor than from other two areas. Comparison between different studies shows that despite many similar trends identified, there are important differences in bioaccessibility among samples and media from different environments.

A comparison between the media shows that in soil, attic, and street dust, the bioaccessibility of individual PTEs is mostly higher in the gastric than in the gastro-intestinal phase. The opposite is true for household dust. General decrease in BAFs in the gastro-intestinal phase of soil, attic, and street dust is a result of changes in physico-chemical conditions during the passage from the stomach to the small intestine. The most important factor is an increase of pH value. Due to the rise in pH from values around 1 to 4 that are typical for the stomach environment to close to neutral values in the small intestine (Ruby et al., 1996), the PTEs mineral phases that was dissolved in stomach can be immobilised by recrystallisation. As oxides, sulphides, and carbonates are usually the most unstable mineral phases in the acidic gastric conditions (Grøn & Andersen, 2003), we assume that the most bioaccessible PTEs in the analysed samples largely occur in these chemical forms. The presence of Cu, Pb and Zn oxides/carbonates and sulphides in original samples was confirmed by SEM/EDS analysis, while no Cd-containing particles were detected (Gaberšek & Gosar, 2021b).

In contrary with soil, attic and street dust, the majority of analysed PTEs in household dust are more bioaccessible in the gastro-intestinal phase. Additionally, there are examples of individual PTEs and samples of soil and attic dust with the same characteristic. An increase of BAFs in the small intestine may be the result of the formation of complexes (e.g. with enzyme pepsin) or the dissolution of PTE-bearing organic matter which can occur at neutral pH in the small intestine and lead to mobilisation of PTEs (Grøn & Andersen, 2003). We assume that higher BAFs in household dust in the gastro-intestinal phase are associated with a significantly higher content of organic matter in this media in comparison to other. The studies of Gaberšek and Gosar (2018, 2021b) determined that organic matter content in household dust in Maribor is approximately 5 times higher than in other media. The analysis with SEM/EDS showed that on average 51% of the surface of household dust samples is covered by organic matter. The predominant influence of higher organic matter content in household dust on its bioaccessibility characteristics was not confirmed by statistically significant correlations between organic matter content and the total levels of PTEs in household dust. This may be due to the insufficient number of analysed samples that would allow us a reliable statistical analysis. Another possible explanation for differences in characteristics of bioaccessibility between household dust and other media may be the result of different origin of PTEs and consequent differences in their chemical/mineral phases which might be less chemically stable in close to neutral pH conditions. The PTEs in household dust predominantly originate from indoor sources and are thus specific for individual apartment and habits of inhabitants, while sources of PTEs of other three media are outdoors (e.g., traffic, industry) (Gaberšek & Gosar, 2021b). Organic matter was recognised as a key factor controlling PTEs bioaccessibility in household dust also by Rasmussen et al. (2008). Wu et al. (2024) confirmed the negative correlations between bioaccessibility of PTEs and pH and particle size of household dust, while a positive correlation was observed with total organic carbon. An important factor is also humidity of the living environment in which the household dust is sampled, since humidity can strongly influence the course of chemical processes and thus the transformation of the primary forms of the elements into other, potentially more mobile chemical species (Zupančič et al., 2020).

### Characteristics of individual solid particles as a key to understand bioaccessibility

Differences in characteristics of bioaccessibility between individual samples of the same medium and especially between household dust and other media may be the result of the different origin of PTEs and differences in their chemical/mineral phases in which PTEs occur in the samples. A detailed characterisation of individual solid particles in various media before and after the in vitro oral bioaccessibility tests is crucial for a more detailed insight into bioaccessibility dynamics. During the extensive geochemical research of urban area of Maribor (Gaberšek & Gosar, 2021b), a detailed characterisation of individual solid particles in all three dust types with the use of SEM/EDS was performed. We identified that Cr, Cu, Ni, Pb, Sb, Sn and Zn appear mostly in the same chemical and mineral phases in all three types of dusts, with only few exceptions. They are often bound in smaller concentrations to different solid particles, such as angular and spherical Fe-oxides, which most often contain Cu and Zn, and to a lesser extent also Cr, Ni, Pb, Sb and Sn. Another particle type containing PTEs is Fe-alloys, of which Fe–Cr (Cu, Mn, Ni) particles are especially common in street dust from the Melje industrial zone. Copper, Pb and Zn occur also as the main constituents of oxide/carbonate, sulphate, and sulphide solid particles. We discovered Cu–Zn shavings in street and attic dust in the Melje industrial zone, originating from the nearby foundry. Cerium and La are mostly bound in the mineral monazite in all dusts. Fe–Ce–La–O spheres appear in household dust in apartments where residents often smoke. We were not able to identify solid carriers of As, Cd and Li with SEM/EDS in neither of the dust type (Gaberšek & Gosar, 2021b). The SEM/EDS analysis did not reveal major differences in the chemical/mineral phases of PTEs, which could explain the differences in bioaccessibility between household dust and other media. The only major difference observed are much higher contents of organic matter in household dust, as already discussed above.

Characterisation of individual solid particles before in vitro oral bioaccessibility test was utilised also by some other studies. Mehta et al. (2019) studied oral bioaccessibility of PTEs (by using UBM) in highly enriched mine waste and soil at abandoned mine sites. With the use of micro-X-ray fluorescence, they determined that the dominant minerals present in soil are clays (kaolinite and montmorillonite), Fe–Al (Mg) silicates, olivine, plagioclase, and pyroxene, and that the secondary minerals are Fe oxides, K-feldspar, Mn phases and sulphides. SEM analysis showed that As, Cr, Cu and Ni were locked within mineral grains. They concluded that bioaccessibility of PTEs strongly depends on soil pH, soil phases, solubility of Fe-rich phases and presence of clay like minerals (Mehta et al., 2019). Kelepertzis et al. (2021) combined study of oral bioaccessibility of PTEs in soil, street dust, and household dust samples using UBM with magnetic measurements and SEM/EDS analyses. They discovered that bioaccessibility of most PTEs in soil and street dust is linked to the magnetic fraction of samples. The SEM/EDS analysis revealed that magnetic particles are presented mainly by the anthropogenic Fe-containing spherules of industrial origin in all sampling media, often containing minor contents of Cr, Cu, Mn, Pb, and Zn. To interpret oral bioaccessibility of Cd, Cu, Pb, and Zn in dust fractions of ores, soils, and technological materials from former mining site, Ettler et al. (2023) also utilised mineralogical and SEM/EDS analyses. Monneron-Gyurits et al. (2024) performed detailed characterisation of soil solid phases before and after bioaccessibility tests to qualify and quantify the phases involved in the mobility of As and Pb to understand the bioaccessibility behaviour by using X-ray diffraction and X-ray Absorption near Edge Structure (XANES). A clear decrease in contents of Pb phases of Pb-goethite, anglesite, and Pb-humate was discovered after the UBM treatment, while no changes were observed in As speciation (Monneron-Gyurits et al., 2024).

**Fig. 3:**
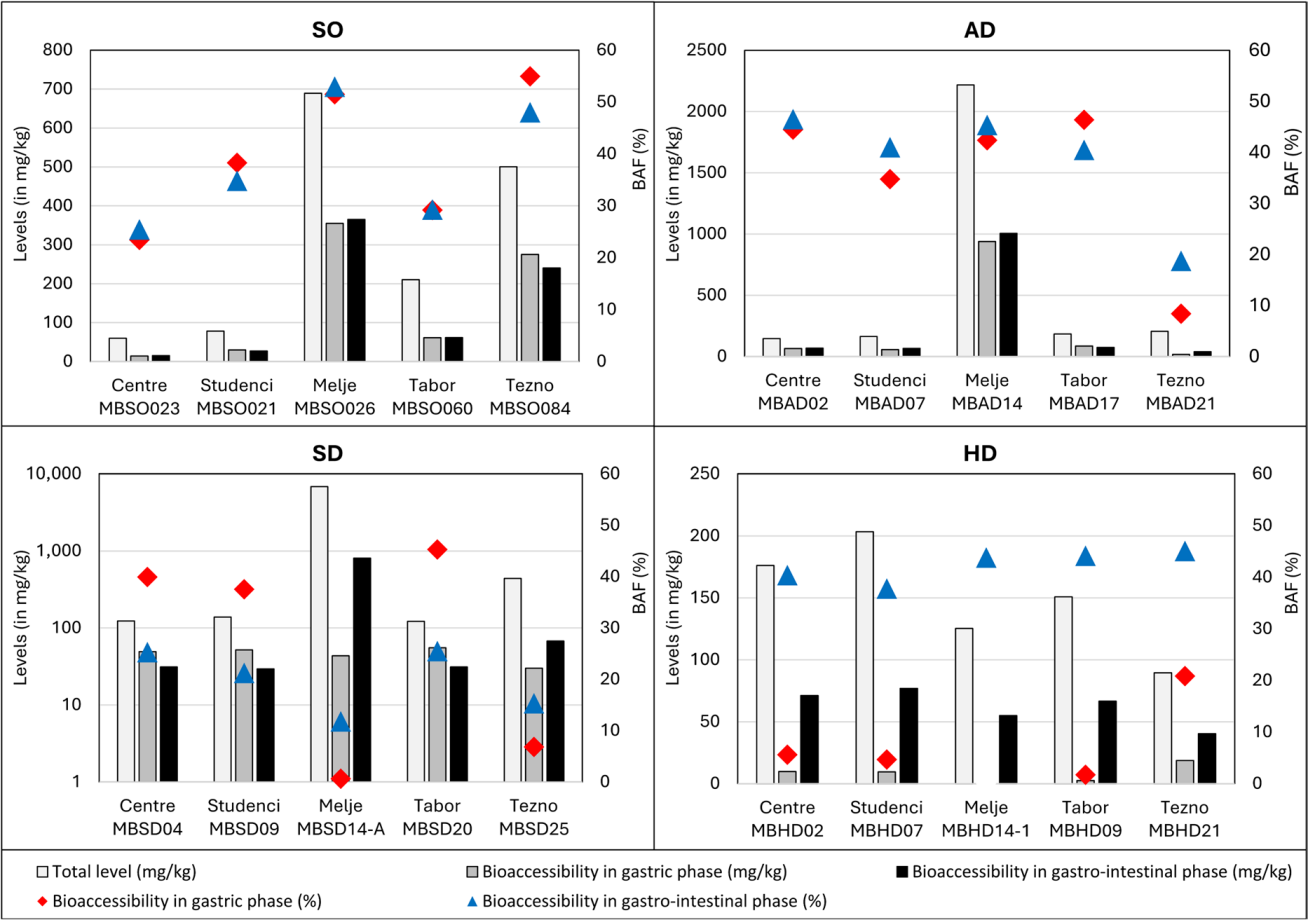
Diagram of total and bioaccessible levels (in mg/kg; columns) and bioaccessible fractions (BAF (%); rhombus-G phase, triangles-GI phase) of Cu in soil, attic dust (AD), street dust (SD), and household dust (HD) (data for AD has been partially published in Gaberšek et al. (2022))

**Fig. 4:**
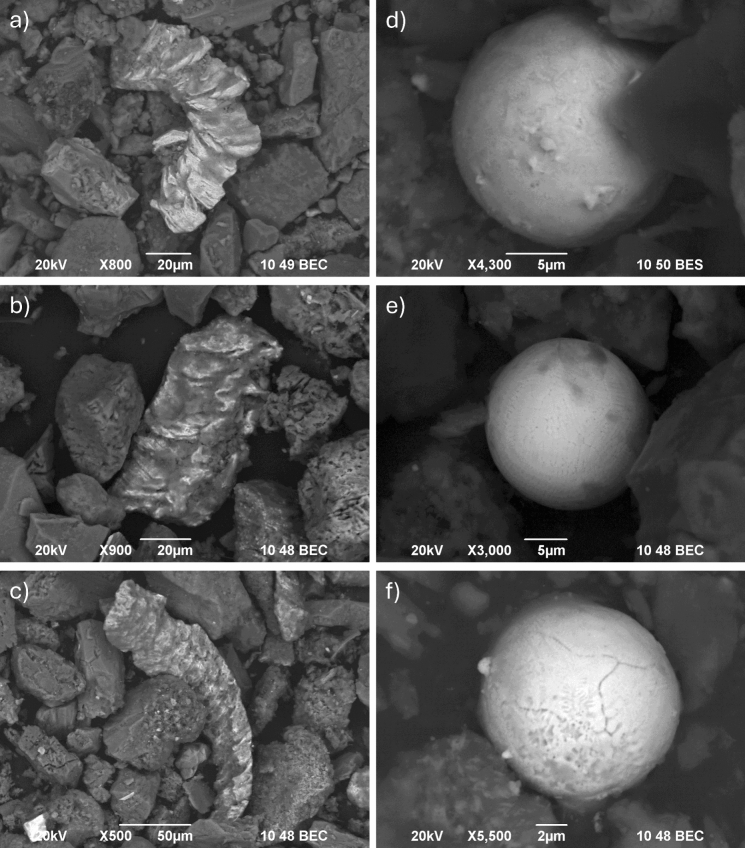
Solid Cu–Zn particles in street dust sample from Melje industrial zone (MBSD14-A) before UBM (**a**), in solid residual of G phase (**b**), and in solid residual of GI phase (**c**), and solid Ce–La–Fe–O spherical particles in household dust sample from Melje industrial zone (MBHD14-1) before UBM (**d**), in solid residual of G phase (**e**), and in solid residual of GI phase (**f**)

As shown above, most of the researchers study the mineralogical, chemical, and morphological characteristics of solid particles in original samples and try to correlate these data with the results of bioaccessibility test. But an important question in bioaccessibility studies is what happens with individual solid particles in human organism and what type of particles contribute the most to the bioaccessible fraction of individual PTEs. That’s why we have done analyses of solid residual of both UBM phases by using SEM/EDS to test the feasibility of this method to address the above questions. We focused on street (MBSD14-A) and household dust (MBHD14-1) samples from the Melje industrial zone. This town’s district is characterised by high total levels of Cu, Pb, and Zn in soil (Gaberšek & Gosar, 2018) and attic and street dust and the presence of Cu–Zn particles, originating from a nearby foundry, and Fe–Cr (Cu, Mn, Ni) shavings, originating from other industrial activities (Gaberšek & Gosar, 2021b). Due to high total levels, the absolute bioaccessible levels of Cu in soil, attic, and street dust in Melje industrial zone are the highest among all samples, with the exception of bioaccessible level in gastric phase of street dust (Fig. 3). On the other hand, corresponding BAFs are not the highest and in the case of street dust BAF is even by far the lowest. We were interested whether the Cu–Zn particles are an important source of bioaccessible Cu. We discovered the same particles with the same morphology and chemical composition within original sample and solid residues of both UBM phases (Fig. 4a–c). That’s why we believe that this particle type is inert in the human digestive tract and that some other form of Cu contributes to the bioaccessible fraction. Based on SEM/EDS analysis, Fe–Cr (Cu, Mn, Ni) particles that are also characteristic for Melje industrial zone also seem chemically stable in the human digestive tract.

**Fig. 5:**
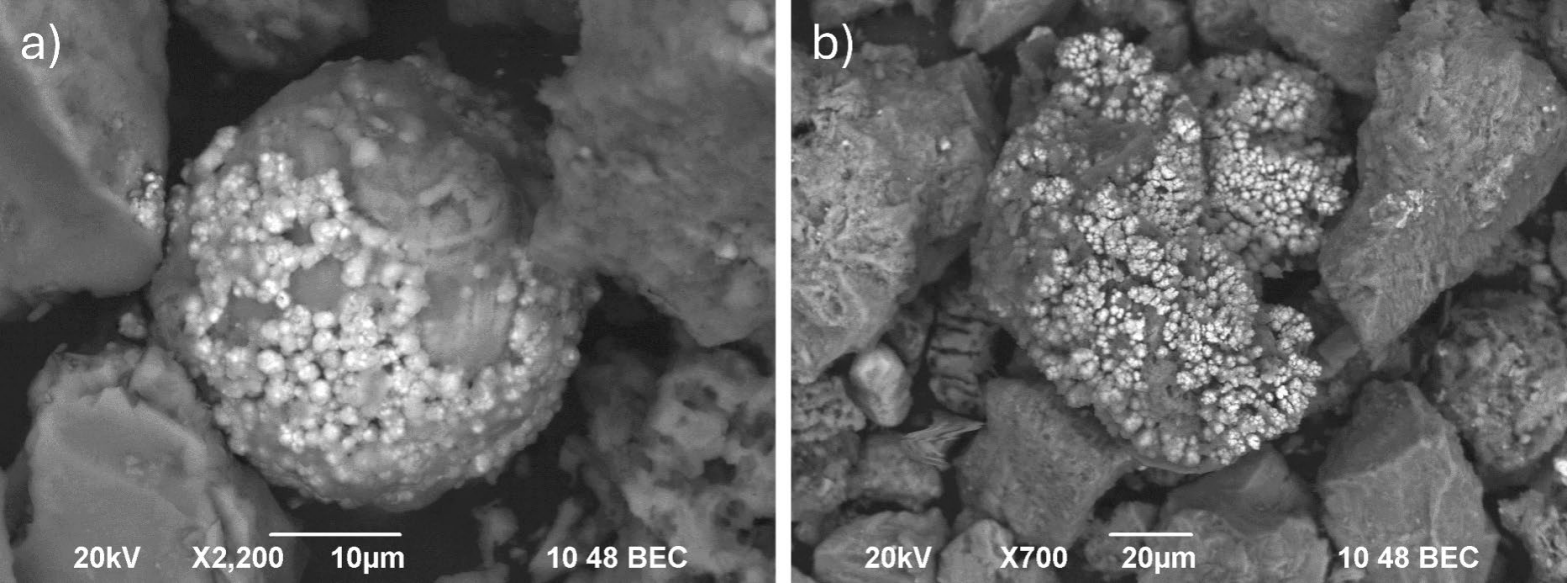
Traces of (re)crystallisation in solid residual of GI phase of street dust sample from Melje industrial zone (MBSD14-A): (**a**) Cu–O (S) crystals (brighter) developed on Fe–Si–O (Al, Mg, Ca) sphere, and (**b**) Cu–O crystals developed on Al–Si–P–O (Cu, Zn, Mn) particle

Interesting solid particles discovered in household dust samples are Fe–Ce–La–O spheres, originating from the use of lighters. Their presence in original sample (MBHD14-1) and in solid residuals of gastric and gastro-intestinal phases (Fig. 4d–f) indicate that they are also chemically stable in case of ingestion. Additionally, we discovered traces of potential precipitation of copper-oxide crystals on the surface of other solid particles (Fig. 5) in residual of gastro-intestinal phase of street dust sample from Melje industrial zone, which is not surprising due the rise of pH in this phase. These preliminary results indicate that scanning electron microscope techniques could contribute significantly to the understanding of oral bioaccessibility.

To the best of our knowledge, the presented research is the first one that analysed chemical and micromorphological characteristics of solid residual of both UBM phases by using SEM/EDS. Nevertheless, similar ideas have been presented recently but they differ in analytical techniques, so direct comparison with our results is limited. For example, Ettler et al. (2019) determined mineral composition of tailing dust, slag dust, and smelter dust samples before and after their exposure to the simulated gastric fluid (SGF) by using X-ray diffraction analysis (XRD). The XRD analysis of the residues after the extraction in SGF indicated changes in the mineral compositions of some samples. Calcite and tennantite almost completely dissolved, while dolomite, willemite, descloizite, and metal carbonates partly dissolved in tailing dust. The most significant changes in mineralogical compositions after extraction were reported for the smelter dusts. Several phases such as gypsum and metal arsenates (johnbaumite, mimetite, alarsite), mullite and gunningite almost completely disappeared and arsenolite was also substantially dissolved, leading to especially high levels of bioaccessible As. They stated that the bioaccessibility of PTEs is highly dependent on the mineralogy (Ettler et al., 2019).

A slightly different approach to the studies of chemical and micromorphological changes at the particle level in case of their ingestion is Differential Individual Particle Analysis (DIPA). DIPA involves the initial characterization of solid particles with SEM/EDS, after which the material is exposed to a liquid (e.g. synthetic digestive fluids) or gas phase reaction for a specified time, and once exposure is concluded, the particles are reanalysed with SEM/EDS and potential chemical and micromorphological changes are determined (Hunt & Johnson, 2011). Bavec et al. (2016) used DIPA to determine the stability of individual PTE-bearing particles in household dust from Hg-polluted area when exposed to simulated stomach acid (SSA). The application of DIPA showed that Hg sulphides (minerals cinnabar and potentially metacinnabar) showed no signs of dissolution in SSA, while for other recognised PTE-bearing particles significant morphological and/or chemical alterations were observed. The most significant changes were observed for solid phases of Pb (Pb oxides/carbonates, Pb sulphates), Zn (Fe oxide/hydroxide with minor Zn and Zn sulphides), Cu (metallic Cu) and Ni (metallic Ni), indicating their high oral bioaccessibility. Similarly, Entwistle et al. (2017) combined UBM and DIPA to determine factors controlling Pb bioaccessibility in contaminated soil. Determined Pb BAFs ranged from < 5% to nearly 90%. The additional analyses with computer-controlled scanning electron microscopy (CCSEM) and DIPA showed that Pb associated with other higher atomic number elements (Fe, Zn, Cu, Ni) was less soluble than when it was present as isolated phases (e.g., as carbonate) or when it was bound with lower atomic number elements (Na, Al, Si, K, Ca). The heterogeneity in solubility and composition of the Pb-particles suggested that the Pb originated from a range of different anthropogenic activities (Entwistle et al., 2017). These examples confirm that identification of micromorphological and chemical alterations at individual particle level is beneficial for understanding the behaviour of solid carriers of PTEs in case of ingestion and to improve our knowledge on dynamics of oral bioaccessibility in general. Our study showed that not only dissolution processes but also the precipitation processes should be considered in such studies.

### Implications for risk assessment

The results of the presented study have important implications for future risk assessment although it did not focus on determining potential health hazard of PTEs. The study confirmed that among the most bioaccessible PTEs in gastric phase are usually Cd, Cu, Pb, and Zn, while in gastro-intestinal are the most bioaccessible Cd, Cu, As, and Ni. The fact that most PTEs are less bioaccessible in small intestine, where the absorption rate of substance into the blood stream is much higher than in stomach, is a good news from a human health point of view. Nevertheless, special attention should be paid to the bioaccessibility in gastro-intestinal phase in the future. This is true especially for the household dust as we spend a lot of time indoors and we are daily exposed to this media. The oral exposure from PTEs in household dust is especially high for children due to their hand-to-mouth activity. The majority of PTEs in household dust showed higher bioaccessibility in gastro-intestinal phase than in gastric phase. These facts together with the higher organic matter content in household dust compared to other media, which is a key factor in the distribution, transformation and thus bioaccessibility of PTEs, require a special attention to be paid to the household dust in risk assessment studies. Another important aspect of the presented study is that several PTEs were included that are usually neglected in health hazard assessment. These PTEs are Ce, La, Li, Sn and also Hg. Their BAFs were relatively low in all samples but hopefully the presented study will encourage researchers to expand the range of PTEs included in bioaccessibility studies, especially the PTEs whose usage is increasing globally. The absolute bioaccessible levels provided in this paper are a good basis for future risk assessment calculations.

## Conclusion

The presented study is the first study that compares oral bioaccessibility of PTEs in four solid media that humans are most often exposed to. These are soil and street, attic, and household dust. Oral bioaccessibility of 13 PTEs was determined, including some that are not very often studied (e.g., Ce, La, Li, Sn) but might also present a human health hazard. As expected, bioaccessibility varies strongly between individual samples of the same medium, between different media, between individual elements and both phases. This is largely the result of differences in mineral and chemical forms in which these elements occur. We confirmed that high total levels of PTEs do not necessarily mean high bioaccessibility and vice versa. Despite all the differences, the most bioaccessible elements in gastric phase are usually Cd, Cu, Pb, Zn, As, and Ni, and Cu, Cd, As, Ni in the gastro-intestinal phase. Due to low pH, bioaccessibility is usually higher in the gastric phase, which is good from the human health point of view, since the absorption of elements from the stomach is lower than from the small intestine. The opposite is true for household dust, in which 11 out of 13 analysed elements have higher bioaccessibility in the gastrointestinal phase. This might be due to much higher organic matter content in this type of dust than in other media and due to presence of PTEs in chemical/mineral phases that are less stable in close-to-neutral conditions of small intestine. As the PTEs in household dust predominantly originate from indoor sources and are thus specific to individual apartment and habits of inhabitants, while sources of PTEs of other three media are outdoors, the differences in their chemical/mineral phases are plausible. The peculiar characteristics of bioaccessibility of PTEs in household dust and our daily exposure to it, requires a special attention to this media in risk assessment studies. Comparison of micromorphological and chemical characteristics of individual solid particles in original samples and in corresponding solid residuals of gastric and gastro-intestinal phases, was proved to be beneficial for understanding the behaviour of solid carriers of PTEs in case of ingestion as well as for improving our knowledge on oral bioaccessibility in general.

### Supplementary Information

Below is the link to the electronic supplementary material.

Supplementary file1 (XLSX 26 KB)
